# Thermal evolution of Andean iron oxide–apatite (IOA) deposits as revealed by magnetite thermometry

**DOI:** 10.1038/s41598-021-97883-3

**Published:** 2021-09-16

**Authors:** Gisella Palma, Martin Reich, Fernando Barra, J. Tomás Ovalle, Irene del Real, Adam C. Simon

**Affiliations:** 1 Department of Geology and Millennium Nucleus for Metal Tracing Along Subduction, FCFM, Universidad de Chile, Plaza Ercilla 803, Santiago, Chile; 2 Andean Geothermal Center of Excellence (CEGA), FCFM, Universidad de Chile, Plaza Ercilla 803, Santiago, Chile; 3 Department of Earth and Environmental Sciences, University of Michigan, 1100 North University Ave, Ann Arbor, MI USA

**Keywords:** Geochemistry, Mineralogy, Geology, Economic geology

## Abstract

Magnetite is the main constituent of iron oxide–apatite (IOA) deposits, which are a globally important source of Fe and other elements such as P and REE, critical for modern technologies. Geochemical studies of magnetite from IOA deposits have provided key insights into the ore-forming processes and source of mineralizing fluids. However, to date, only qualitative estimations have been obtained for one of the key controlling physico-chemical parameters, i.e., the temperature of magnetite formation. Here we reconstruct the thermal evolution of Andean IOA deposits by using magnetite thermometry. Our study comprised a > 3000 point geochemical dataset of magnetite from several IOA deposits within the Early Cretaceous Chilean Iron Belt, as well as from the Pliocene El Laco IOA deposit in the Chilean Altiplano. Thermometry data reveal that the deposits formed under a wide range of temperatures, from purely magmatic (~ 1000 to 800 °C), to late magmatic or magmatic-hydrothermal (~ 800 to 600 °C), to purely hydrothermal (< 600 °C) conditions. Magnetite cooling trends are consistent with genetic models invoking a combined igneous and magmatic-hydrothermal origin that involve Fe-rich fluids sourced from intermediate silicate magmas. The data demonstrate the potential of magnetite thermometry to better constrain the thermal evolution of IOA systems worldwide, and help refine the geological models used to find new resources.

## Introduction

Magnetite (Fe_3_O_4_) forms under a wide range of pressure and temperature conditions in igneous, sedimentary and metamorphic environments, as well as in a variety of magmatic and hydrothermal ore deposits. Geochemical studies of magnetite from a wide range of mineral deposits have demonstrated that physico-chemical parameters such as temperature, pressure and oxygen fugacity (ƒO_2_) control its minor and trace element budget, particularly for Ti, V, Mn, Cr, Mg and Al^1–7^. Therefore, the geochemical composition and microtextural features of magnetite provide key information that helps elucidate the formation environment and the chemical evolution of magnetite-bearing deposits^5,8–16^.

Magnetite is the main constituent of iron oxide–apatite (IOA) ore deposits, commonly referred to as Kiruna-type deposits, which can host hundreds of millions to several billion tonnes of magnetite. The microtextures and trace element and isotopic compositions of magnetite samples from Andean IOA deposits have been widely studied^10,11,15,17–28^. These studies scaffold a framework to better understand the ore-forming processes, revealing that the Fe ore forms by mechanisms that involve growth of magnetite under a wide range of conditions, spanning from high-temperature, purely igneous settings, to lower-temperature, fluid-dominated hydrothermal environments. Despite these advances, the thermal evolution of IOA deposits remains poorly constrained and, to date, obtaining quantitative temperature data directly from magnetite mineralization has been challenging. This raises uncertainties on genetic models proposed to explain the genesis of Andean IOA deposits, which over the years have invoked: (1) immiscible Fe-, P-, and carbonate-sulfate-rich melts^29–33^; (2) metasomatic replacement^17,34,35^; and (3) magmatic-hydrothermal processes^10,11,21,24,25,36,37^. Temperature estimations for Andean IOA deposits have been determined using oxygen isotope thermometry between magnetite-actinolite and magnetite-pyroxene pairs^19,23,24,27,29^, thermometry of magnetite-ilmenite pairs^38^, actinolite thermometry based on Fe contents^19,22,27^, and fluid and melt inclusion thermometry in apatite, pyroxene, quartz, anhydrite and calcite^11,33,39–42^. However, temperature data obtained directly from magnetite in these deposits are either scarce or unavailable.

**Figure 1:**
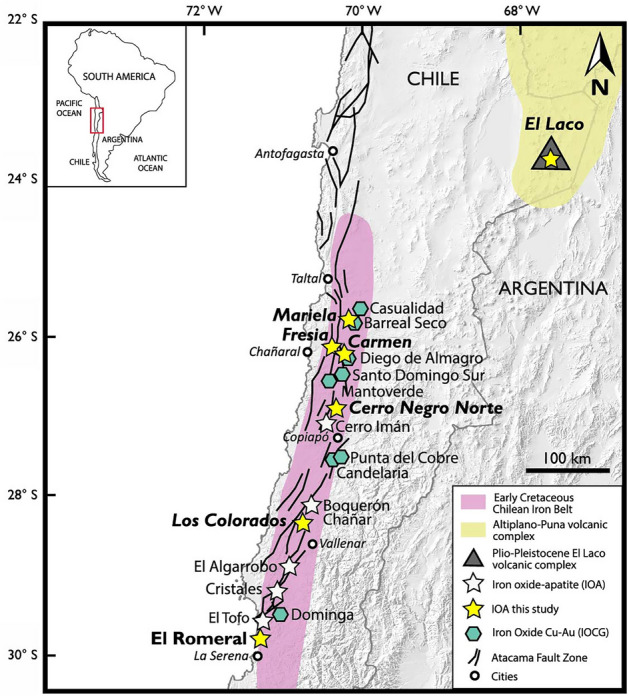
Location of the main iron oxide–apatite (IOA) deposits within the Early Cretaceous Chilean Iron Belt, and the Pleistocene El Laco volcanic complex in the Chilean Altiplano. Modified from Palma et al.^26^.

In this study, we use the trace element concentration of magnetite to provide a quantitative estimation of temperature during the evolution of Andean IOA deposits. We apply the Canil and Lacourse^43^ magnetite thermometer (T_Mg-mag_), which is based on Mg and Fe concentrations, to a comprehensively compiled magnetite EMPA and LA-ICP-MS database. The temperature data were obtained from > 3000 analyses of magnetite from IOA deposits in the world-class Early Cretaceous Chilean Iron Belt, and the Pliocene El Laco deposit in the Central Andes (Fig. 1). We also tested the applicability of the magnetite thermometer by coupling temperature determinations with trace element data (Ti, V and Ga) and micron- to nano-scale observations of magnetite grains in each deposit. This allowed us to confirm the robustness of this thermometer, as well as elucidating micro-analytical uncertainties that lead to temperature overestimations and potential misinterpretations.

## Sources of data and methods

A total of 3126 EMPA and LA-ICP-MS spot analyses of magnetite from different Andean IOA deposits (Table S1) were compiled and used for thermometry calculations. The samples were collected from several deposits in the Early Cretaceous Chilean Iron Belt, including Los Colorados ^10,11,44^, El Romeral^22,26^, Cerro Negro Norte^28^, Carmen, Fresia and Mariela^26^, as well as the Pliocene Laco Norte and Laco Sur magnetite bodies^21,25^. In each deposit, the cited studies have recognized a variety of magnetite types based on their microtextures and chemical composition^26^. Magnetite textures vary from pristine and inclusion-free, to grains that contain abundant mineral inclusions, Fe-Ti lamellar exsolutions, oscillatory and sector zoning, symplectite, and dissolution, reequilibration and recrystallisation textures. These textural features provide insights into magmatic, hydrothermal and syn- and post-mineralization reequilibration processes, as well as a general assessment of the temporality of the different magnetite types. For the sake of simplicity, and to maintain consistency with the magnetite parageneses described in the literature, we homogenized the terminology of the main magnetite textural types according to temporality, from the earliest- to the latest-formed, i.e., *Mgt-1, -2, -3, -4* (Table S2). Detailed descriptions for each deposit and their magnetite types can be found in the Supplemental Material (Appendix 1, 2).

Temperature calculations were carried out using the T_Mg-mag_ thermometer by Canil and Lacourse^43^, which is based on the observation that X_Mg_ = [Mg/(Mg + Fe_total_)] in magnetite is strongly dependent on temperature. These authors experimentally demonstrated that the Mg concentration in magnetite is more dependent on temperature, and relatively insensible to ƒO_2_ changes, than other trace elements (Al, Mn, Cr, Ni) for a wide range of *P–T–X*–ƒO_2_ conditions, in both magmatic and hydrothermal environments. Hence, temperatures were calculated here by using the Fe and Mg concentrations of magnetite determined by EMPA and LA-ICP-MS (Tables S1), and the following empirical calibration, which considers an uncertainty of ± 50 °C^43^:

$${T}_{Mg-mag}(^\circ \mathrm{C})= - \frac{8344 (\pm 322)}{ln{X}_{Mg}-4.13 (\pm 0.28)}-273$$

The calculated temperature data were coupled with trace element data (Ti, V, Ga) and magnetite temporality based on microtextural observations. Typically, these elements are enriched in igneous magnetite and their concentrations increase systematically with increasing temperature^1–5^. This allowed us to differentiate between the multiple magnetite types in each deposit. Based on textural relations and trace element concentrations, a decreasing temperature (T) trend is expected from the earliest to the latest magnetite generations (e.g., T_*Mgt-1*_ > T_*Mgt-2*_ > T_Mgt-3_ > T_*Mgt-4*_). A statistical summary of Ti, V and Ga concentrations and calculated temperatures are presented in Table S3.

In addition to magnetite from Cretaceous IOA deposits, we determined crystallisation temperatures (T_Mg-mag_) for two Fe orebodies (Laco Norte and Laco Sur) and the andesitic host rock from the Pliocene El Laco deposit using published data^4,20^. This allowed us to have a direct temperature reference of the igneous magnetite to compare with temperatures obtained for magnetite from the orebodies using the T_Mg-mag_ thermometer.

## Results

**Figure 2:**
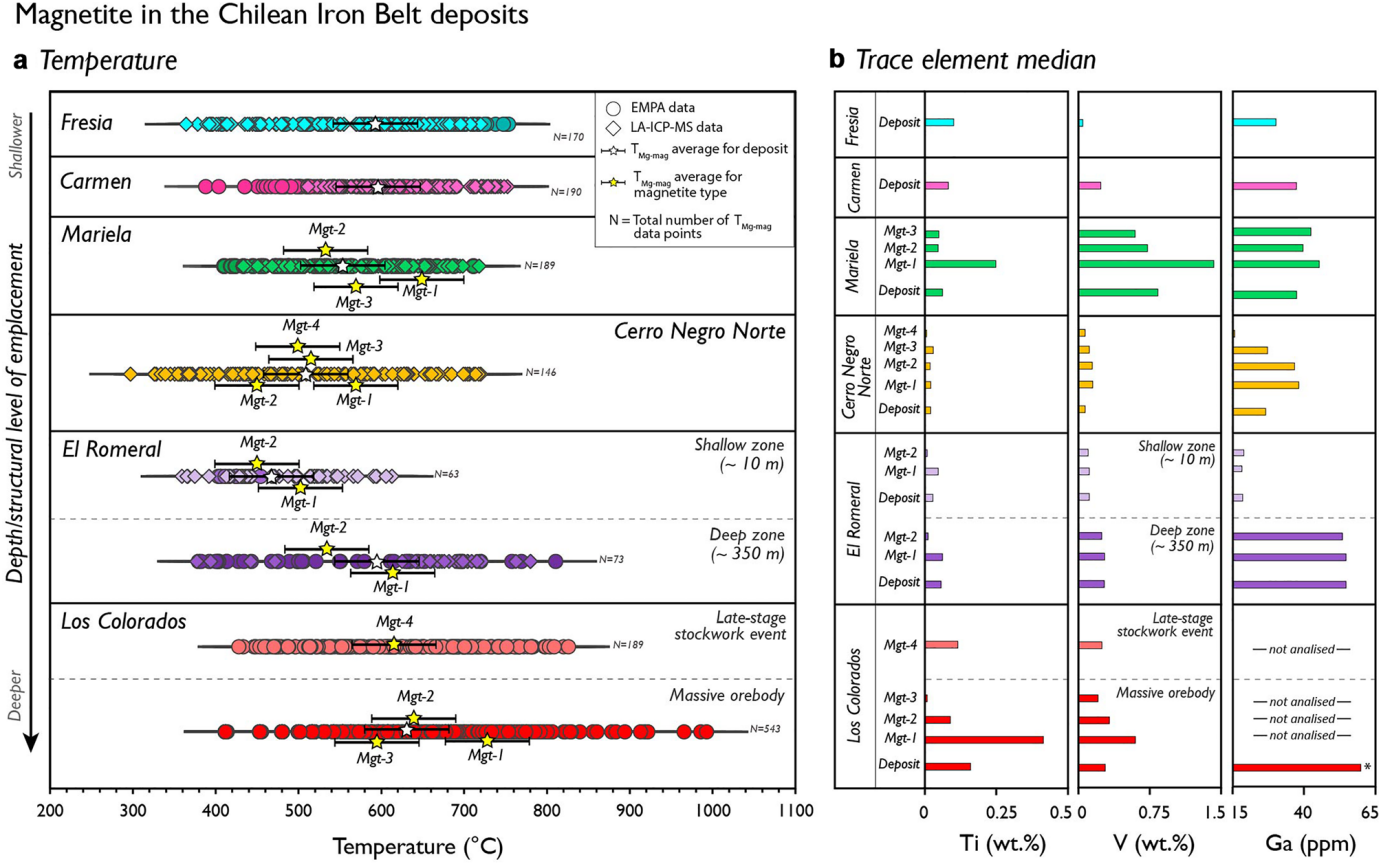
Calculated temperatures and median concentrations of selected trace elements in magnetite from IOA deposits of the Chilean Iron Belt. **(a)** Temperatures calculated using the T_Mg-mag_ thermometer^43^. Uncertainties (error bars) are at ± 50 °C. **(b)** Titanium, V and Ga median concentrations determined by LA-ICP-MS, except for Los Colorados in which EMPA (Ti, V) and LA-ICP-MS (Ga) data were used.

**Figure 3:**
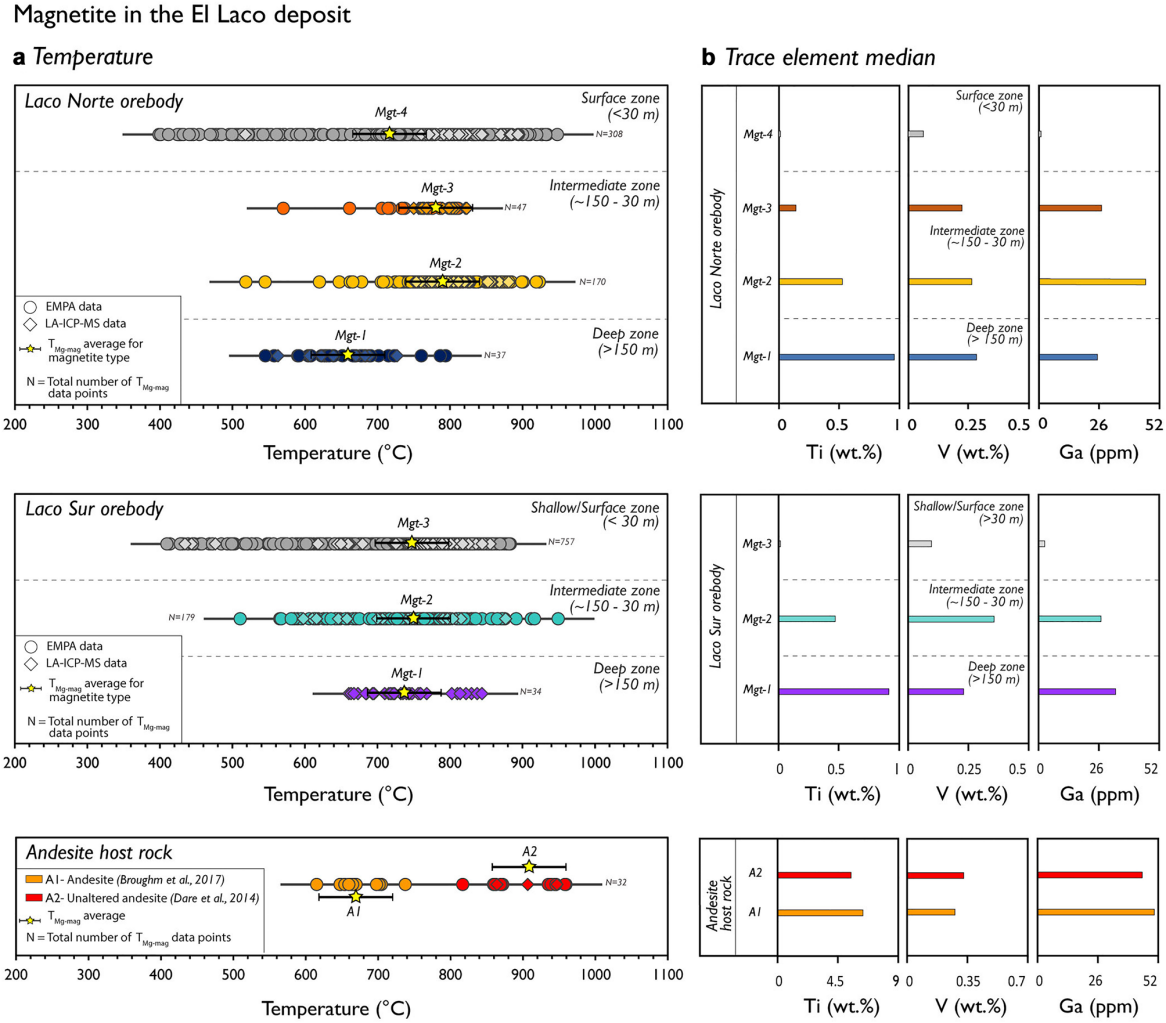
Calculated temperatures and median concentrations of selected trace elements in magnetite from El Laco orebodies and its andesitic host rocks. **(a)** Temperatures calculated using the T_Mg-mag_^43^ thermometer. Uncertainties (error bars) in T_Mg-mag_ are at ± 50 °C. **(b)** Titanium, V and Ga median concentrations determined by LA-ICP-MS.

Calculated magnetite crystallisation temperatures for IOA deposits at the Chilean Iron Belt and El Laco are plotted in Figs. 2a and 3a, respectively. In addition, the median concentrations of Ti, V and Ga in magnetite are plotted for the same deposits (Figs. 2b, 3b). Detailed microtextural data are available for most deposits, including paragenetic relations among magnetite types (temporality), as well as observable variations with depth (Table S2). However, in a few cases (e.g., Carmen and Fresia), no clear temporality between magnetite types has been reported.

Figure 2a shows the calculated temperatures for magnetite orebodies in the Chilean Iron Belt. Crystallisation temperatures for magnetite from the Los Colorados IOA deposit mostly range between ~ 850 and ~ 500 °C, with *Mgt-1*, *Mgt-2*, *Mgt-3* and *Mgt-4* configuring a cooling trend with averages values of ~ 730 °C, ~ 630 °C, ~ 600 °C and ~ 620 °C, respectively. Calculated temperatures for El Romeral magnetite vary from ~ 780 to 380 °C in the *deep zone* to ~ 600 to 340 °C in the *shallow zone*. In both zones, paragenetically early *Mgt-1* yields higher average temperatures than late *Mgt-2*. In Cerro Negro Norte, temperatures range from ~ 700 to ~ 320 °C, and the average temperatures for the different magnetite generations (*Mgt-1* to *Mgt-4*) range from ~ 570 to ~ 450 °C. Magnetite from Mariela yields temperatures that range from ~ 720 to ~ 420 °C, and an average temperature of ~ 650 °C for *Mgt-1,* ~ 520 °C for *Mgt-2* and ~ 590 °C for *Mgt-3*. Finally, magnetite from the Carmen and Fresia deposits yield a similar range of calculated temperatures, i.e., ~ 750 to 440 °C and ~ 750 to 360 °C, respectively, and a similar average temperature of ~ 600 °C for both deposits.

Temperatures for the Laco Norte and Laco Sur orebodies at the El Laco deposit mostly range between ~ 900 and ~ 400 °C (Fig. 3a). The temperature range for the *deep*, *intermediate* and *shallow zones* are similar for both orebodies. For the three zones and the different magnetite types (*Mgt-1* to *Mgt-4*), the average calculated temperatures range between ~ 700 °C and ~ 800 °C. The *shallow/surface zone* shows a wide dispersion with calculated temperatures between ~ 940 and ~ 400 °C. Temperatures for magnetite in the andesite host rocks, on the other hand, range between ~ 960 and ~ 620 °C, and the average temperatures are between ~ 900 °C and ~ 670 °C (Fig. 3a, lower panel).

## Discussion

### Temperature trends in Andean IOA deposits

The temperature data obtained for magnetite from IOA deposits within the Chilean Iron Belt reveal a distinct cooling trend that broadly correlates with the relative depth of formation or structural level of emplacement of the deposits^26^. The deep, intrusive-type Los Colorados deposit has the highest calculated temperatures (~ 850–500 °C; Fig. 2a). Intermediate temperatures (~ 780–340 °C) characterise the transitional El Romeral deposit, followed by lower temperatures (~ 700–320 °C) in the hydrothermal-type Cerro Negro Norte deposit (Fig. 2a). Interestingly, temperatures for early magnetite (*Mgt-1*) within each deposit are consistently higher than the other late textural types in most deposits (Fig. 2a). In Los Colorados, El Romeral and Mariela, this decreasing temperature trend correlates with trace element concentrations, most notably Ti, V and Ga, which are higher in *Mgt-1* (Fig. 2b).

In Los Colorados, the high magnetite temperatures are consistent with oxygen isotope thermometry data (> 850 °C to ~ 610 °C), and re-homogenization temperatures of polycrystalline inclusions (> 950 °C) in *Mgt-1*^11,19^. In addition, the core-to-rim temperature gradient—T_*Mgt-1*_ (~ 730 °C) > T_*Mgt-2*_ (~ 630 °C) > T_*Mgt*-3_ (~ 600 °C)—agrees with the qualitative magnetite cooling path determined by using the [Ti + V] vs. [Al + Mn] concentration plot^3,5,9^ (Fig. S1). The latter was interpreted by Knipping et al.^10,11^ as the result of cooling from high-temperature, magmatic-hydrothermal (> 600 °C) to lower temperature hydrothermal conditions (< 600 °C). Notably, hydrothermal magnetite (*Mgt-4*) from the *late-stage stockwork event* at Los Colorados^44^ yields a similar average temperature (~ 620 °C; Fig. 2a) to those determined for the late-stage rims; *Mgt-2* and *Mgt-3* (~ 630 and 600 °C; Fig. 2a), formed over *Mgt-1* cores in the massive magnetite orebodies.

Magnetite temperature data for El Romeral, reveal that the Ga concentration can be used to discriminate between magnetite from the high-temperature *deep zone* and the lower-temperature *shallow zone* (Figs. 2, S2). Furthermore, temperatures from the *deep zone* correlate well with those determined by the composition of primary actinolite grains at 1 kbar (~ 805 to ~ 735 °C)^22^. In Cerro Negro Norte, the average temperature for early *Mgt-1* is consistently higher (~ 572 °C), although data for the other textural types (*Mgt-2 to -4*) cluster within average temperatures of ~ 520 to 450 °C (Fig. 2a). Pegmatitic-type IOA deposits such as Mariela, Carmen and Fresia (~ 750 to 360 °C; Fig. 2a) exhibit evidence of widespread dissolution-reprecipitation processes, which have been interpreted as caused by multiple pulses of both magmatically-derived and externally-derived meteoric fluids and/or basinal brines^26,45^. Fluid inclusion studies by Velasco and Tornos^42^ at Carmen are consistent with this notion, suggesting the involvement of aqueous-gas rich, high-salinity (> 30 wt.% NaCl eq) Ca–Cl–Mg fluids with temperatures > 360 ºC. Average crystallisation temperatures for magnetite from Carmen and Fresia (~ 600 °C; Fig. 2a) are consistent with oxygen isotope thermometry temperatures determined for coeval magnetite-actinolite pairs in Carmen^42^.

Temperature data for magnetite from El Laco andesitic host rocks provide further insights into the conditions of magnetite crystallisation from a silicate melt. Magnetite in the unaltered host andesite^4^, which is undoubtedly of igneous origin^20,29^, crystallised at higher temperatures (~ 960–820 °C) than magnetite from the orebodies (~ 900–400 °C; Fig. 3a). Magnetite samples from the Laco Norte and Laco Sur orebodies at El Laco yield crystallisation temperatures that are similarly variable (Fig. 3a). Furthermore, no major temperature variations are observed with depth or with the temporality of magnetite types in these orebodies. Despite the relatively high average calculated temperature of magnetite from the *shallow/surface zone* (up to ~ 940 °C), a lower temperature “tail” (~ 400–350 °C) that corresponds with a depletion in Ti-V-Ga is observed (Fig. 3a,b). Previous studies reported high temperatures at El Laco as documented by fluid inclusion thermometry. Temperatures of ~ 840 to ~ 700 °C and high salinities (0.2–59 wt.% NaCl eq.) were obtained from fluid inclusions in clinopyroxene and apatite intergrown with magnetite^39,40^. Recently, Bain et al.^33^ reported liquid–vapor homogenization temperatures between ~ 951 and 800 °C for polycrystalline inclusions in diopside-magnetite-anhydrite veins from the El Laco Pasos Blancos orebody. These temperatures overlap with those estimated from oxygen isotope thermometry in the diopside-magnetite-anhydrite veins (~ 1125 to 900 °C)^29^.

### Thermal evolution and genetic implications

**Figure 4:**
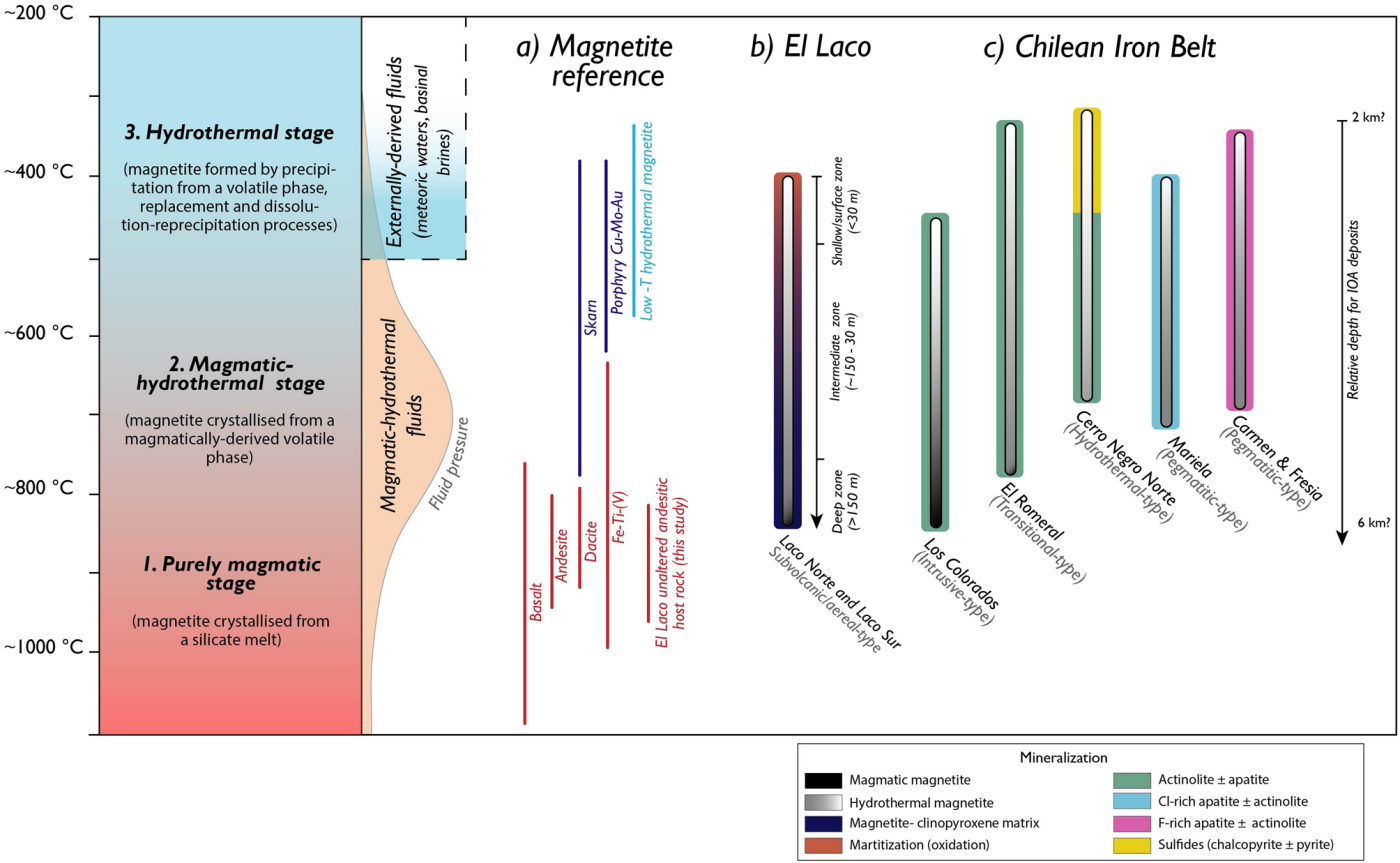
Thermal evolution of Andean IOA deposits unraveled by magnetite thermometry data. *Stages 1* and *2* are characterised by *purely magmatic* (~ 1000 to 800 °C) and *magmatic-hydrothermal* (~ 800 to 600 °C) temperature conditions, respectively. S*tage 3* comprises purely *hydrothermal* (< 600 °C) conditions. **(a)** Reference temperatures of magnetite for igneous rocks (basalt, andesite, dacite), and magmatic/magmatic-hydrothermal (Fe–Ti, V, skarn, porphyry Cu–Mo–Au) ore deposits, and low-temperature (T) hydrothermal magnetite (replacement and disseminated); **(b,c)** thermal evolution of El Laco **(b)** and IOA deposits from the Chilean Iron Belt **(c)**. See text for discussion.

Figure 4 presents a schematic representation of magmatic-hydrothermal stages illustrating the thermal evolution for Andean IOA deposits. We coupled the magnetite thermometry data reported here with reference temperatures calculated for magnetite crystallisation in igneous rocks (basalt, andesite, dacite) and other magmatic/magmatic-hydrothermal ore systems including Fe-Ti, V deposits, skarn and porphyry Cu-Mo-Au deposits^20,46,47^ (Figs. 4a, S3; Table S4). The new temperature data for magnetite presented here are consistent with a magmatic-hydrothermal origin for Andean IOA deposits. The magnetite thermometry data agree with δ^56^Fe and δ^18^O stable isotope data for magnetite in several IOA deposits in Chile that are consistent with magnetite crystallising from high-temperature fluids sourced from silicate melts^18,19,23,24,34,37,48^. Furthermore, the data correspond well with ^56^Fe and ^18^O isotope information for several other IOA deposits worldwide, formed under different tectonic settings in different epochs. These include IOA deposits in the Kiruna and Gräsbergerg districts in Sweden, the Bafq District, Iran, and the Pea Ridge and Pilot Knob deposits in Missouri, USA, among others^23,48–50^.

Our proposed model in Fig. 4 invokes a combination of magmatic and hydrothermal processes to explain the thermal evolution of IOA deposits in continental arc settings. *Stage 1* comprises purely igneous magnetite crystallisation from a hydrous silicate melt, reflected by temperatures of ~ 1000 to 800 °C. During this stage, upward migration of magma to 3–4 km depth^26^ results in decompression-induced volatile saturation of the silicate melt, facilitated by heterogenous nucleation onto magnetite microlites^10,11,51–53^. Decompression allows an efficient separation of the Fe-rich magmatic volatile phase from the parental magma reservoir and its rapid transfer to upper levels through faults, forming tabular, massive magnetite bodies such as those found at Los Colorados^10,26^. During *stage 2* (~ 800–600 ºC), the dissolved FeCl_2_ in the exsolved magmatic-hydrothermal fluid precipitates as magnetite upon cooling, which is more efficient under higher degrees of decompression^22,54^. Mineralization styles include stockwork and breccias, typical of systems dominated by high water/rock ratios (e.g., El Romeral, Cerro Negro Norte, *deep/intermediate zones* of El Laco). *Stage 3* involves magnetite precipitation from cooling hydrothermal fluids at temperatures below 600 °C, with variable contributions from available external fluids, e.g., meteoric fluids and/or basinal brines^23,55^. During this stage, multiple injections of fluids lead to complex magnetite growth, with increased dissolution-reprecipitation processes and formation of pegmatitic bodies and pervasive replacement horizons, e.g., Carmen, Fresia, Mariela, El Laco^21,26,45^.

The thermal trends recorded in magnetite in the studied orebodies suggest that Andean IOA deposits were formed predominantly under high-temperature magmatic-hydrothermal (~ 800–600 °C) conditions that grade to lower temperature hydrothermal conditions (< 600 °C; Fig. 4b,c). These results agree with most recent studies in IOA systems^18,19,23,24,26,37^ and similarly to other magnetite-bearing magmatic-hydrothermal deposits such as skarns and porphyry Cu-Mo-Au systems^8,9,16,46^ (Fig. 4a).

### Considerations for magnetite thermometry in ore systems

The crystallisation temperatures reported in this study, calculated from a database of over 3000 magnetite analyses, are unequivocally consistent with geological observations and independent temperature estimations in the studied deposits, confirming the robustness of the magnetite thermometer. We highlight that the proper application of the T_Mg-mag_ thermometer depends not only on a good experimental and empirical calibration^43^, but also relies on detailed textural studies, including identification of chemical zonation, exsolution, Mg-bearing nano- to micro-sized inclusions and domains, as well as oxidation products (hematite, maghemite, goethite). These features are frequently observed and particularly affect magnetite in ore deposits due to the significant hydrothermal and chemical weathering overprinting in these systems. Therefore, it is likely that some of the temperatures calculated in this study may be representative of multiple episodes of hydrothermal circulation, leading to higher average temperatures. For example, magnetite from the intermediate zone in El Laco^21^ yields high calculated temperatures of up to ~ 940 °C (Fig. 3a), possibly due the abundance of micron- to nano- scale Mg-bearing silicate particles (Table S2). Thus, it is likely that hydrothermal processes such as fluid-aided dissolution-reprecipitation lead to variations in the calculated temperatures attributable to complex magnetite textures in some deposits (e.g., El Laco, Carmen, Fresia). This may result in an overestimation of the Mg concentration in magnetite and consequently higher calculated temperatures, where the average Mg concentration in magnetite corresponds to the sum of Mg in the magnetite matrix and silicate nano-inclusions^44^. Additionally, variable degrees of magnetite low-temperature oxidation and replacement by maghemite, hematite and goethite—typically found at shallow levels at El Laco (*Mgt-3* and*-4;* Table S2)—could explain the wide range of magnetite temperatures (~ 950–390 °C Fig. 3a). Accordingly, relatively high-Mg and low-Ti, V, Ga contents of these magnetite grains (Fig. 3b) reflect extensive chemical modification.

## Final remarks

We reconstructed the thermal evolution of Andean iron oxide–apatite (IOA) deposits by using the T_Mg-mag_ thermometer on a large magnetite geochemical dataset. Our results are the first comprehensive assessment of the thermal evolution of IOA deposits, providing a quantitative estimation of cooling trends in several IOA deposits of variable size and types. Calculated magnetite temperatures record a transition from purely igneous (~ 1000 to 800 ºC) to mainly hydrothermal conditions (< 600 °C). Our data support a genetic model that invokes a magmatic-hydrothermal origin for Andean IOA deposits, and most importantly, reveal a predominance of fluid-dominated hydrothermal conditions. Our results demonstrate that magnetite thermometry opens new avenues to constrain formation temperatures in IOA systems, and therefore could be useful for vectoring towards magnetite-rich zones laterally and vertically, and for inferring the presence of deeper mineralized orebodies.

## Methods

Several analytical techniques were used to characterise the textures and quantify the composition of magnetite from massive orebodies in Andean IOA deposits^10,11,21,22,25,26,28,44^, and only a brief description is presented here. Identification and characterisation of magnetite textures, as well as the selection of sample sites for EMPA and LA-ICP-MS analyses, were performed by using a scanning electron microscope (SEM). Electron probe microanalyses (EMPA) and laser ablation inductively coupled plasma spectrometry (LA-ICP-MS) were used to quantify the abundances of major (e.g., Mg, Al, Si, Ca, Ti, V, Cr, Fe, Mn) and trace elements (e.g., Na, Mg, Al, Si, P, Ca, Cr, Mn, Co, Ni, Cu, Zn, V, Ti, Ga) in magnetite samples, respectively. The complete database of magnetite compositions (Fe, Mg, Ti, V, Ga) is provided in Table S1.

Crystallisation temperatures of magnetite from Andean IOA deposits were obtained by using the T_Mg-Mag_ thermometer^43^, which is applicable to both igneous and hydrothermal magnetite. Calculations were performed using the empirical calibration: T_Mg-Mag_ (°C) = –8344(± 322)/[lnX_Mg_ – 4.1 (± 0.28)] – 273, in which X_Mg_ = Mg/(Mg + Fe_total_), and considering an uncertainty of ± 50 °C.

## Supplementary Information

Supplementary Tables.

Supplementary Information.

## Data Availability

The authors declare that the data supporting this study are available within the paper and its supplementary information**.**
