# Supplementary material for: Thermal evolution of Andean iron oxide–apatite (IOA) deposits as revealed by magnetite thermometry
Source: Sci Rep. 2021 Sep 16;11:18424. doi: 10.1038/s41598-021-97883-3 (PMC8445919; doi:10.1038/s41598-021-97883-3)
Supplement: Supplementary file 2 — Supplementary Information. [file 41598_2021_97883_MOESM2_ESM.pdf]

Supplementary Material for:

Thermal evolution of Andean Iron Oxide-Apatite (IOA) deposits as revealed by magnetite thermometry

Gisella Palma, Martin Reich, Fernando Barra, J.Tomás Ovalle, Irene del Real, Adam C. Simon

*Corresponding author: gipalma@ing.uchile.cl

This file includes:

Excel files:

- Tables S1 to S4

This pdf file:

- Appendix 1. Geologic Background and IOA deposit subtypes
- Appendix 2. Microtextures and geochemistry of studied magnetite generations
- Figures S1 to S3

Appendix 1. ***Geologic Background and IOA deposit subtypes***

Los Colorados, El Romeral, Cerro Negro Norte, Carmen, Fresia and Mariela IOA deposits are located within the Early Cretaceous Chilean Iron Belt in the Coastal Cordillera of Northern Chile (Fig. 1). Within this belt, IOA and IOCG deposits are usually spatially and temporally associated (Fig. 1). Both types of deposit are commonly hosted by volcanic rocks of intermediate-mafic composition, occur closely associated with coeval magmatism and are structurally controlled by the arc-parallel Atacama Fault System (Sillitoe, 2003; Williams et al., 2005). Magnetite bodies vary from massive, veins, mantos and breccias, with variable amounts of actinolite, apatite and traces of sulfides such as pyrite and chalcopyrite. Magnetite samples from each deposit were collected throughout the length of drill cores that traverses massive magnetite ore bodies from Los Colorados, El Romeral and Cerro Negro Norte (Knipping et al., 2015a,b; Rojas et al., 2018; Salazar et al., 2020), or from stockpiles (Carmen and Fresia) and abandoned underground mine (Mariela) (Palma et al., 2020).

Each IOA deposit in the Chilean Iron Belt represents specific formation conditions that range from magmatic to hydrothermal to pegmatitic IOA mineralization, which are consistent with different emplacement levels within the upper crust (Palma et al., 2020). Los Colorados, El Romeral and Cerro Negro Norte massive bodies are mainly composed of a magnetite-actinolite assemblage. Smaller pegmatitic-type deposits including the Mariela breccia pipe and vein-type Carmen and Fresia are characterized by a magnetite-apatite ± actinolite assemblage with a ~20–40% modal apatite and represent the upper section of IOA systems and formation under high volatile/melt conditions (Treloar and Colley, 1996; Palma et al., 2019, 2020).

The El Laco volcanic complex (23° 48´ S) is located at 4600-5200 m above sea level in the Chilean altiplano (Fig.1). The El Laco ore deposit represents a shallow, subvolcanic/aereal-type of IOA deposits (Palma et al., 2020), and is probably one of the best-exposed examples of magnetite mineralization directly linked to volcanic activity on a continental arc stratovolcano (Ovalle et al., 2018). This deposit comprises a cluster of magnetite orebodies (Laco Norte, Laco Sur, San Vicente Alto, San Vicente Bajo, Rodados Negros and Rodados Grandes, Pasos Bancos and Extesion Laco Sur) emplaced around the central Pico Laco volcanic edifice. In this contribution, we focused on texturally and chemically well-characterized magnetite samples from two of the main iron oxide orebodies (Laco Norte and Laco Sur), and accessory magnetite from the andesite volcanic units that host the magnetite ore (Ovalle et al., 2018; La Cruz et al., 2020). The andesite samples were collected from outcropping volcanic units in Pico Laco dome, San Vicente Bajo and Cristales Grandes.

Appendix 2. ***Microtextures and geochemistry of studied magnetite generations***

Magnetite microtextural types include pristine/inclusion-free, inclusion-rich, Fe-Ti lamellar exsolutions, symplectite, reequilibration and recrystallization textures. Mineral inclusions, single to polycrystalline of micron- to nano-size, are randomly distributed in magnetite cores, trace-element rich bands (e.g., oscillatory zoning and colloform banding) and/or zones (e.g., sector zoning) or are arranged following crystallographic planes or display oscillatory and sinuisal patterns (Palma et al., 2020). The main textural features and a statistical summary of the trace element concentrations (Ti, V, Ga) of the different magnetite generations for each deposit are summarized in Table S2 and Table S3.

At Los Colorados, three magnetite generations (*Mgt-1, -2, -3*) form part of the massive ore body (Knipping et al., 2015a,b) and a fourth generation (*Mgt-4)* of the late hydrothermal brecciated/stockwork zone (Deditius et al., 2018). At El Romeral, two magnetite generations (*Mgt-1* and *Mgt-2*) were identified in both the *deep zone* (~350 m) and *shallow zone* (~10 m) (Rojas et al., 2018b; Palma et al., 2020), while the Cerro Negro Norte ore body was formed by four generations of magnetite (*Mgt-1, -2, -3, -4*; Salazar et al., 2020) (Table S2). In these three deposits, *Mgt-1* (inclusion-rich cores) and *Mgt-2* (inclusion-poor to pristine rims/overgrowths) are the most relevant and

abundant magnetite types (>90% modal). In terms of chemical composition of magnetite in these deposits, the Los Colorados deposit shows the highest Ti, V and Ga average concentration, followed by El Romeral and Cerro Negro Norte (Table S3). Similarly, the trace element composition of magnetite gradually decreases from earliest to latest magnetite generations in Los Colorados (i.e., *Mgt-1* > *Mgt-2* > *Mgt-3*) and Cerro Negro Norte (i.e., *Mgt-1* > *Mgt-2* > *Mgt-3* > *Mgt-4*) and is contrasting (notably Ga) between *deep* (higher Ti, V, Ga) and *shallow zone*s (lower Ti, V, Ga) from El Romeral (Table S3). Massive magnetite from Mariela occurs in close association with ilmenite, rutile, titanite and ulvöspinel phases and comprises three main magnetite generations (Palma et al., 2020). *Mgt-1* and *Mgt-2* display ilmenite/ulvöspinel oxy-exsolution lamellae and symplectite-like textures, respectively, whereas *Mgt-3* corresponds to late veinlets/overgrowths related to dissolution-reprecipitation processes (Table S2). Otherwise, we do not rename magnetite generations of Carmen and Fresia deposits due dissolution-reprecipitation textures (e.g., porosity, sharp replacement fronts) obliterated primary textures (e.g., oscillatory zoning, colloform banding, sector zoning) and the chemical composition of magnetite (Tables S2, S3). Therefore, no geochemical trends are observed between different magnetite generations (Palma et al., 2020). Magnetite from Mariela shows relatively higher Ti, V and Ga concentrations, particularly V, in comparison to Carmen and Fresia (Table S3).

El Laco orebodies are vertically zoned and develop complex depth-dependent microtextural and geochemical gradients (Ovalle et al., 2018; La Cruz et al., 2020). Broadly, the Laco Sur orebody displays a more intense hydrothermal alteration than Laco Norte. The *deep zone* (>150 m depth) is dominated by magnetite-rich breccias composed of Ca-Na-hydrothermally altered volcanic clasts and a magnetite-clinopyroxene (± ilmenite ± scapolite ± titanite) matrix. Magnetite at depth is characterized by ilmenite oxy-exsolution lamellae (*Mgt-1*; >150 m depth; Table S2), that occur along with ilmenite grains. The *intermediate zone* (~150-30 m) corresponds to a breccia body-massive magnetite transitional zone characterized by magnetite grains with diverse microtextural features, including dissolution-reprecipitation textures, variable degrees of oxidation, oscillatory- and sector-zoning (Table S2). The sinuous and resorbed magnetite cores display sector zoning with inclusion-rich areas and/or oscillatory zoning and resorption textures, while magnetite overgrowths are characterized by an oscillatory pattern (*Mgt-2* ~ 65-150 m; Table S2). At shallow levels (< 65 m) magnetite grains are mainly characterized by a weakly to moderate hematite replacement (*Mgt*-3; Table S2). The *surface/shallow zone* (~30 m-surface) comprise the main part of the massive orebody, and includes highly vesicular magnetite (>90% modal magnetite) with minor diopside and apatite. Magnetite aggregates (*Mgt-4),* which are moderately to pervasively oxidised to hematite, goethite and maghemite, also display oscillatory-and-sector zoning, reequilibration and recrystallization textures (Table S2). Magnetite grains from andesites occur as microphenocrysts within the groundmass and form magnetite-pyroxene±apatite glomerocrysts and sparse aggregates. Magnetite microtextures vary from pristine, ilmenite oxy-exsolution lamellae to highly porous magnetite with clinopyroxene occurring towards the grain rim. Remarkably, Ti, V and Ga concentrations decrease systematically in accessory magnetite in the andesite host rock > deep zone magnetite > intermediate zone magnetite> magnetite from the shallow/surface zone (Table S3).

Figures Supplementary Material

Figure S1. (a) Reference [Ti+V] vs. [Al+Mn] plot from Nadoll et al. (2014) after Dupuis & Beaudoin (2011). The plot shows different fields, i.e., *Fe-Ti, V*; *Kiruna*, *porphyry*, *skarn*, *IOCG* and *BIF*, which reflect relative conditions for magnetite formation. The arrow reflects the transition from high-temperature, *purely magmatic* (red), to intermediate, *magmatic-hydrothermal* (orange) to low-temperature, *hydrothermal* (blue) conditions for magnetite formation. (b) [Ti+V] vs. [Al+Mn] plot of the different magnetite generations (*Mgt-1, 2 and 3*) of the massive orebody from Los Colorados, colored according to calculated temperatures using the $T_{Mg\text{-}mag}$ geothermometer.

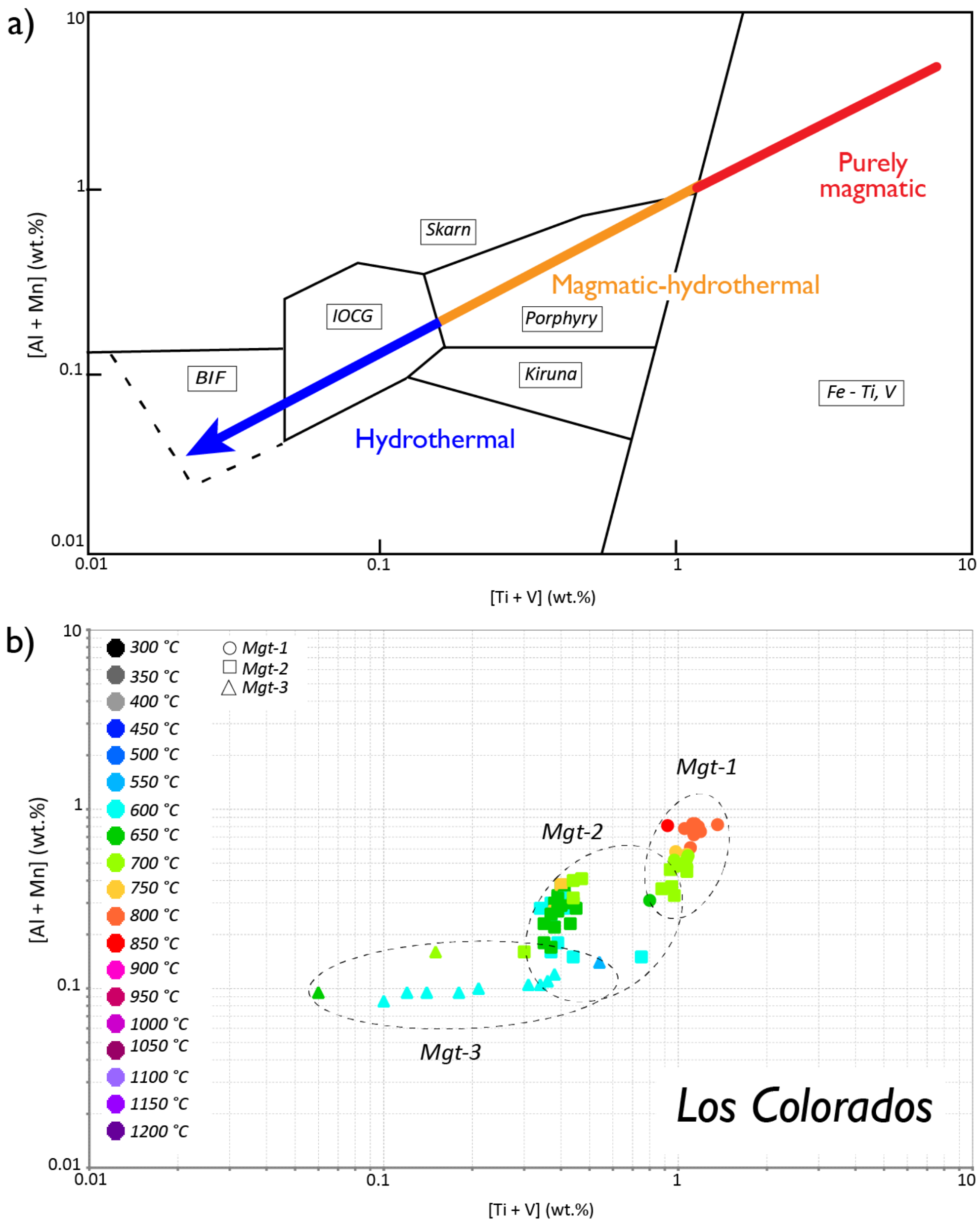

Figure S2. Temperature versus Ti, V and Ga biplots for magnetite types from El Romeral. Note that Ga clearly discriminates between the high-temperature *deep zone* and the low-temperature *shallow zone*.

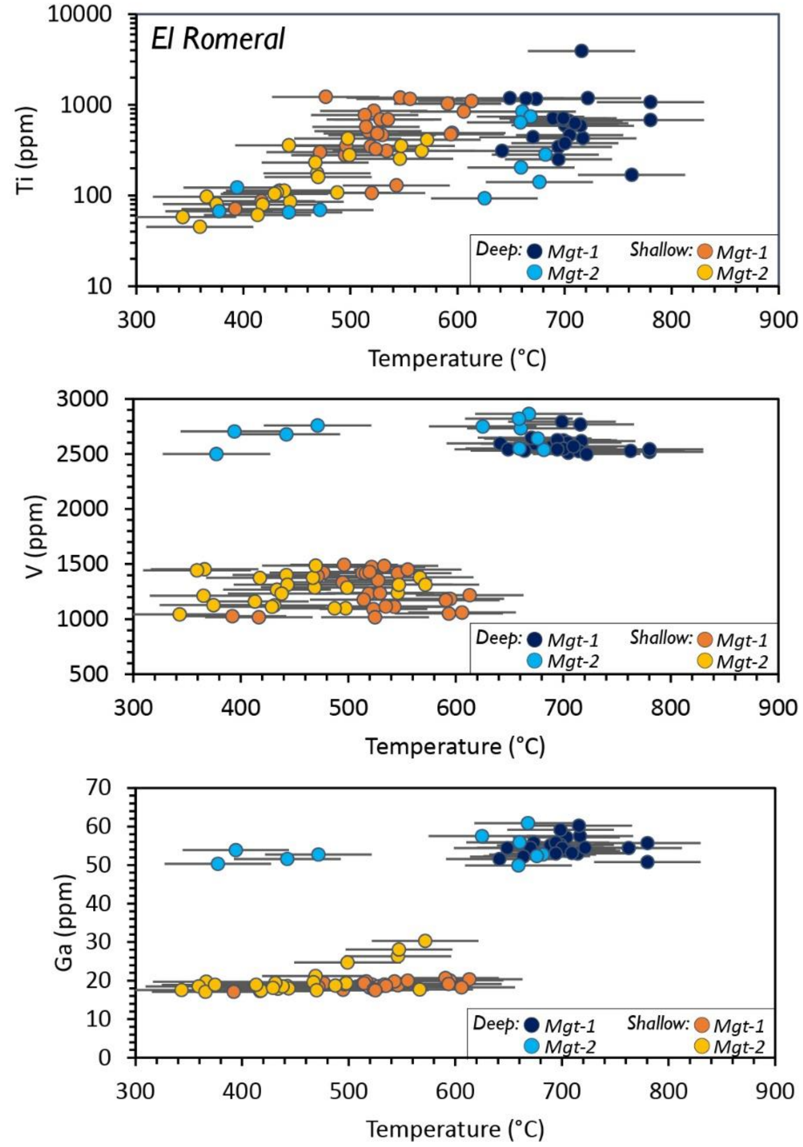

Figure S3. Estimated average temperature ($T_{Mg\text{-}mag}$) calculated for accessory magnetite in volcanic rocks (basalt, andesite, dacite), Fe-Ti, V orthomagmatic, and magmatic-hydrothermal (skarn, porphyry) ore deposits, as well as low-temperature hydrothermal magnetite. Data source: Nadoll (2011), Liu et al. (2015), Broughm et al. (2017), Wen et al. (2017 and references therein).

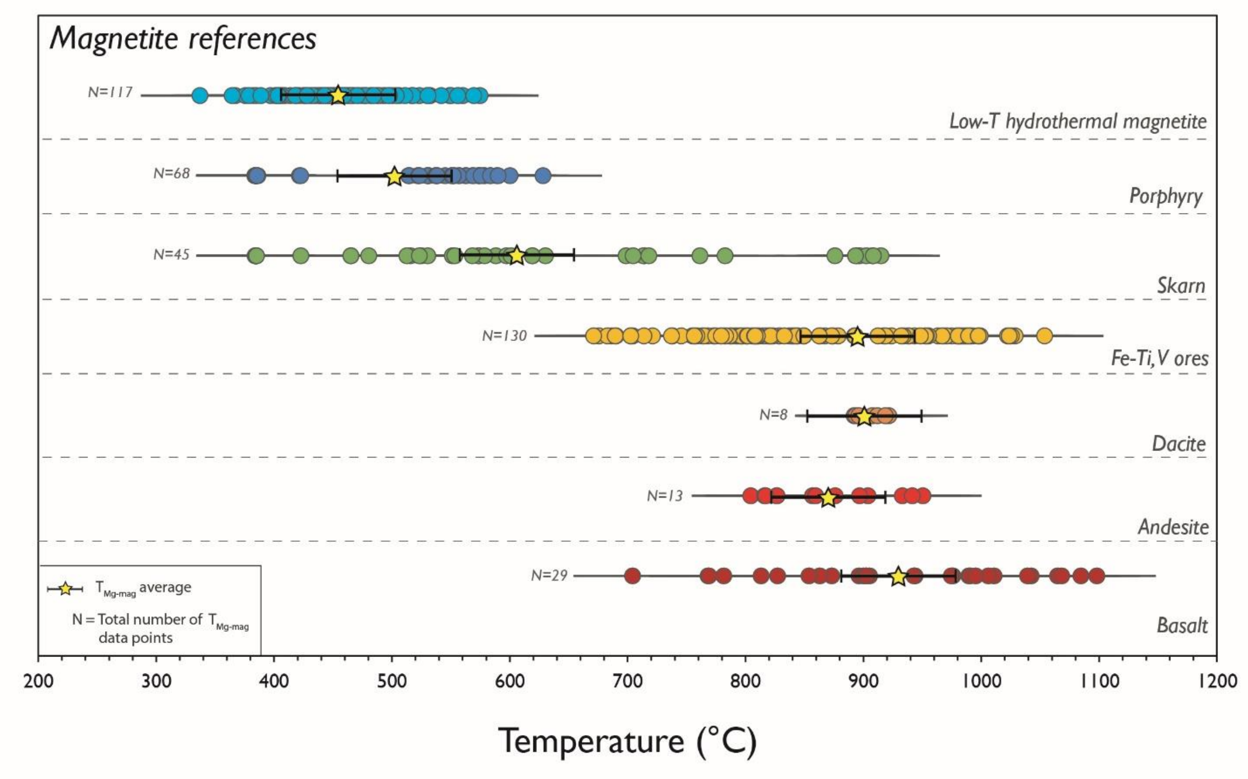

References Supplementary Material

Broughm, S. G., Hanchar, J. M., Tornos, F., Westhues, A. & Attersley, S. Mineral chemistry of magnetite from magnetite-apatite mineralization and their host rocks: Examples from Kiruna, Sweden, and El Laco, Chile. *Miner. Deposita*, **52**, 223–244 (2017).

Deditius, A. P. *et al*. Nanogeochemistry of hydrothermal magnetite. *Contrib. Miner. Petrol.* **173**, 46 (2018).

Dupuis, C. & Beaudoin, G., 2011. Discriminant diagrams for iron oxide trace element fingerprinting of mineral deposit types. *Miner. Deposita* **46**, 319-335.

Knipping, J. L. *et al*. Giant Kiruna-type deposits form by efficient flotation of magmatic magnetite suspensions. *Geology* **43**, 591-594 (2015a).

Knipping, J. L. *et al*. Trace elements in magnetite from massive iron oxide-apatite deposits indicate a combined formation by igneous and magmatic-hydrothermal processes. *Geochim. Cosmochim. Acta* **171**, 15-38 (2015b).

La Cruz, N. L. *et al*. The geochemistry of magnetite and apatite from the El Laco iron oxide-apatite deposit, Chile: Implications for ore genesis. *Econ. Geol.* **115**, 1461–1491(2020).

Liu, P. P., Zhou, M. F., Chen, W. T., Gao, J. F. & Huang, X. W. In situ LA-ICP-MS trace elemental analyses of magnetite: Fe-Ti-(V) oxide-bearing mafic-ultramafic layered intrusions of the Emeishan Large Igneous Province, SW China. *Ore Geol. Rev*. **65**, 853–871 (2015).

Nadoll, P. Geochemistry of magnetite from hydrothermal ore deposits and host rocks - case studies from the Proterozoic Belt Supergroup, Cu-Mo-porphyry + skarn and Climax-Mo deposits in the western United States. Ph. D. thesis, University of Auckland (2011).

Nadoll, P., Angerer, T., Mauk, J. L., French, D. & Walshe, J. The chemistry of hydrothermal magnetite: a review. *Ore Geol. Rev*. **61**, 1-32 (2014).

Ovalle, J. T. *et al*. Formation of massive iron deposits linked to explosive volcanic eruptions. *Sci. Rep.* **8**, 14855 (2018).

Palma, G., Barra, F., Reich, M., Simon, A.C. & Romero, R. A review of magnetite geochemistry of Chilean iron oxide-apatite (IOA) deposits and its implications for ore-forming processes. *Ore Geol. Rev.* **126**, 103748 (2020).

Rojas, P. *et al*. New contributions to the understanding of Kiruna-type iron oxide-apatite deposits revealed by magnetite ore and gangue mineral geochemistry at the El Romeral deposit, Chile. *Ore Geol. Rev.* **93** 413-435 (2018).

Salazar, E. *et al*. Trace element geochemistry of magnetite from the Cerro Negro Norte iron oxide−apatite deposit, northern Chile. *Miner. Deposita* **55**, 409-428 (2020).

Sillitoe, R. Iron oxide–copper–gold deposits: An Andean view. *Miner. Deposita* **38**, 787–812 (2003).

Wen, G., *et al*. Hydrothermal reequilibration of igneous magnetite in altered granitic plutons and its implications for magnetite classification schemes: Insights from the Handan-Xingtai iron district, North China Craton. *Geochim. Cosmochim. Acta* **213**, 255-270 (2017).

Williams, P. J. *et al*. Iron oxide-copper-gold deposits: Geology, space-time distribution, and possible modes of origin. *Econ. Geol. 100th Anniv. Vol*, 371–406 (2005).
